# Stochasticity, heterogeneity, and variance in longevity in human populations

**DOI:** 10.1016/j.tpb.2017.01.001

**Published:** 2017-04

**Authors:** Nienke Hartemink, Trifon I. Missov, Hal Caswell

**Affiliations:** a University of Amsterdam, Netherlands; b Max Planck Institute for Demographic Research, Germany; c University of Rostock, Germany; d Woods Hole Oceanographic Institution, United States

**Keywords:** Individual stochasticity, Heterogeneous frailty, Variance, Longevity, Age–frailty classified matrix model, ​Gamma-Gompertz–Makeham

## Abstract

Inter-individual variance in longevity (or any other demographic outcome) may arise from heterogeneity or from individual stochasticity. Heterogeneity refers to differences among individuals in the demographic rates experienced at a given age or stage. Stochasticity refers to variation due to the random outcome of demographic rates applied to individuals with the same properties. The variance due to individual stochasticity can be calculated from a Markov chain description of the life cycle. The variance due to heterogeneity can be calculated from a multistate model that incorporates the heterogeneity. We show how to use this approach to decompose the variance in longevity into contributions from stochasticity and heterogeneous frailty for male and female cohorts from Sweden (1751–1899), France (1816–1903), and Italy (1872–1899), and also for a selection of period data for the same countries.

Heterogeneity in mortality is described by the gamma-Gompertz–Makeham model, in which a gamma distributed “frailty” modifies a baseline Gompertz–Makeham mortality schedule. Model parameters were estimated by maximum likelihood for a range of starting ages. The estimates were used to construct an age×frailty-classified matrix model, from which we compute the variance of longevity and its components due to heterogeneous frailty and to individual stochasticity. The estimated fraction of the variance in longevity due to heterogeneous frailty (averaged over time) is less than 10% for all countries and for both sexes. These results suggest that most of the variance in human longevity arises from stochasticity, rather than from heterogeneous frailty.

## 1 Introduction

Individual variance, especially in fitness components, plays a key role in demography, ecology, and evolutionary biology. From an evolutionary perspective, variance in fitness components is potential material on which natural selection can operate. From a demographic perspective, identifiable differences among individuals are the basis for structured population models (Metz and Diekmann, 1986, Tuljapurkar and Caswell, 1997, Caswell, 2001); differences due to age lead to age-structured models, differences due to size lead to size-structured models, etc.

Longevity (age at death) is a fitness component that varies widely among individuals. This variance arises as a result of two different underlying causes: individual stochasticity and heterogeneity. Individual stochasticity is variance due to random outcomes of probabilistic demographic processes (living or dying, reproducing or not, making or not making a life cycle transition). Even in a completely homogeneous population, in which every individual experienced exactly the same (age-specific) mortality rates, variance due to individual stochasticity would exist (Caswell, 2009). Any calculation of the variance in longevity from an ordinary life table implicitly assumes that every individual is subject to the (age-specific) mortality rates in that life table, and hence that the variance is only due to individual stochasticity.

Variance in longevity can also result from unobserved, or latent, heterogeneity in the properties of individuals. For example, individuals of the same age may differ in their mortality rates due to genetic, environmental, or maternal effects. Such differences are often referred to as heterogeneity in individual frailty (Vaupel et al., 1979). Because more frail individuals are more at risk than others, heterogeneity in frailty leads to changes in cohort composition with age, due to within-cohort selection. As a cohort ages, the representation of less frail individuals increases, and the average mortality rate in an old cohort will be lower than one would expect based on extrapolation of mortality rates at younger ages. This selection effect has been suggested as an explanation for the mortality plateaus often observed at very old ages (Horiuchi and Wilmoth, 1998, Vaupel et al., 1979, Vaupel, 1985).

The effects of unobserved heterogeneity in survival analysis can be estimated using frailty models (Vaupel et al., 1979, Wienke, 2010). In frailty models, a baseline mortality schedule is modified by a term representing individual frailty. A widely used example is the gamma-Gompertz model, which assumes an exponentially increasing age-specific baseline mortality rate (the Gompertz model), and that frailty acts as a proportional hazard multiplier of the baseline mortality (Vaupel et al., 2014). Frailty, which is fixed over the life of the individual, follows a gamma distribution, the variance of which measures the amount of unobserved heterogeneity.

The variance in longevity in a frailty model is a result of both stochasticity and heterogeneity. Little is known about the relative contribution of each to the total variance in longevity, and how those contributions may depend on species, sex, environmental conditions, etc. Caswell (2014) presented an ad hoc approach to this problem, using an age × frailty-classified matrix model. The variance in longevity was computed from the model and the relative contributions of heterogeneity and stochasticity estimated by reducing the initial variance in frailty to zero and attributing the remaining longevity variance to stochasticity. In an analysis of gamma-Gompertz parameters for a single year Swedish females (obtained from Missov, 2013), the fraction of variance due to heterogeneity was estimated to be only 0.071. Applying the same approach to a gamma-Gompertz–Makeham model for women from Turin (Zarulli et al., 2013) resulted in an even lower estimate of 0.012.

Here, we present a more rigorous variance decomposition, which does not require a hypothetical reduction of frailty variance to zero. We apply it to large cohort mortality data sets for three different countries: Sweden, France and Italy, over a long time period. This will enable us to see whether any patterns in variance can be generalized across countries, time periods, or sexes.

The paper is organized as follows. Section  2 describes the gamma-Gompertz–Makeham mortality model. Section  3 presents the construction of the age × frailty matrix model, and Section  3.2 provides the methods used to calculate longevity statistics and decompose the variance. Section  4 gives details about the mortality data and estimation of the gamma-Gompertz–Makeham parameters. Section  5 presents the implementation of the age × frailty matrix model. Section  6 presents the results for the cohort and period data, and Section  7 discusses the interpretation of the results.

## 2 Frailty in the gamma-Gompertz–Makeham model

Unobserved heterogeneity in mortality risk, or frailty, can be included in mortality models by assuming that this frailty acts to modify a baseline mortality rate shared by all individuals. The gamma-Gompertz–Makeham mortality model has been shown to give a good fit to human mortality data (Manton et al., 1986, Yashin et al., 1994). It is an extension of the Gompertz model (Gompertz, 1825), in which mortality at adult and older ages is an exponentially increasing function of age. The Gompertz mortality function has a baseline mortality parameter a and a parameter b that determines the steepness of the exponential increase with age. The Makeham model an age-independent component c to the mortality (Makeham, 1860). The Makeham term has been shown to be essential to prevent distorted parameter estimates (Missov and Németh, 2016). In the Gompertz–Makeham model, the hazard at age x equals:

(1) $$\mu \left(x\right)=ae^{bx}+c\text{.}$$

In the gamma-Gompertz–Makeham model (hereafter called ΓGM), the frailty of an individual is included as a (gamma-distributed) random effect that is fixed over the lifetime. Dynamic frailty, that can change with age or with health-related events, has been included in other models (e.g.,  Vaupel and Yashin, 2006, Le Bras, 1976, Gavrilov and Gavrilova, 1991, Yashin et al., 1994, Yashin et al., 2000). The matrix analysis we develop here also applies to such dynamic frailty models (Caswell, 2014); see Section  3.1. Frailty in the ΓGM affects the (age-dependent part of) mortality as a proportional hazard; the hazard μ(x,z) for an individual with frailty z at age x is

(2) $$\mu \left(x,z\right)=zae^{bx}+c\text{.}$$

The initial frailty distribution in the cohort is gamma-distributed, Z∼Γ(κ,λ), with shape parameter κ and scale parameter λ. The mean and variance of this distribution are E(Z)=κ/λ and $V\left(Z\right)=\kappa /\lambda^{2}$. The mean is set equal to 1, so that the cohort starts life with an average frailty of 1. When this is the case, i.e. E(Z)=1, λ=κ and the variance V(Z)=1/λ≔γ. The marginal hazard function, which gives the unconditional population hazard Manton et al. (1981) and Missov and Vaupel (2015), is a sigmoid function

(3) $$\mu \left(x\right)=\frac{ae^{bx}}{1+\frac{a\gamma }{b}\left(e^{bx}-1\right)}+c\text{.}$$

Heterogeneity is described by the variance γ of frailty at the starting age of analysis; the higher the variance, the greater the heterogeneity between individuals. In a completely homogeneous population, V(Z)=0 and every individual experiences the same age-dependent hazard.

Using (3) and applying maximum likelihood yields estimates for the baseline mortality parameters a, b, c and for γ, the parameter that describes the heterogeneity in frailty. This optimization is described in more detail in Section  4.2.

## 3 An age×frailty matrix model

We incorporated the ΓGM mortality function into an age × frailty-classified matrix model (for a more general description of age–stage classified matrix models see Caswell, 2009, Caswell, 2012). Age is described by a set of ω discrete age classes and frailty by a set of g frailty classes that discretize the gamma distribution of frailty. Vector $\mu_{0}$ of dimension ω contains the baseline age-specific part of the mortality rates:

(4) $$\mu_{0}=\left(\begin{array}{c} ae^{0b}\\ \vdots \\ ae^{\left(\omega -1\right)b} \end{array}\right)\text{.}$$

If $z_{i}$ is the frailty for the ith group, then the mortality vector for frailty group i is

(5) $$\mu_{i}=z_{i}\mu_{0}+c\;i=1,\ldots ,g\text{.}$$

### 3.1 Cohort projection

The state of the cohort at age t is given by a vector $\overset{˜}{n}\left(t\right)$, which is derived from an array

(6) $$N\left(t\right)=\left(\begin{array}{ccc} n_{11} & \cdots & n_{\omega 1}\\ \vdots & & \vdots \\ n_{1g} & \cdots & n_{\omega g} \end{array}\right)$$

that describes the abundance of all age–frailty categories. The age–frailty population vector is

(7) $$\overset{˜}{n}=\mathrm{vec}\;N\text{;}$$

that is, (8) The jth block of entries in $\overset{˜}{n}$ contains a sub-vector giving the abundance of the g frailty classes within age class j.

The survival of frailty class i is given by a survival matrix $U_{i}$ of dimension ω×ω that contains age-specific survival probabilities on the first subdiagonal and zeros elsewhere.

(9) $$U_{i}=\left(\begin{array}{cccc} 0 & 0 & \cdots & 0\\ e^{-\mu \left(z_{i},0\right)} & 0 & \cdots & 0\\ \vdots & \ddots & & \vdots \\ 0 & \cdots & e^{-\mu \left(z_{i},\omega -1\right)} & 0 \end{array}\right)\text{.}$$

The transition probabilities among frailty classes for age class j are given by a matrix $D_{j}$, of dimension g×g. However, in the ΓGM model, frailty is fixed, so $D_{j}=I_{g}$ for all j. In a model with dynamic frailty, $D_{j}$ would be a column-stochastic matrix of frailty class transition probabilities. Caswell (2014, Section 5.4) gives an example where frailty develops as a diffusion process with reflecting boundaries.

Block-diagonal matrices U and D are created by placing the $U_{i}$ (respectively, $D_{j}$) on the diagonal with zeros elsewhere. Both matrices are of dimension ωg×ωg.

(10) $$U=\left(\begin{array}{ccc} U_{1} & \cdots & 0\\ \vdots & \ddots & \vdots \\ 0 & \cdots & U_{g} \end{array}\right)\;D=\left(\begin{array}{ccc} D_{1} & \cdots & 0\\ \vdots & \ddots & \vdots \\ 0 & \cdots & D_{\omega } \end{array}\right)\text{.}$$

The joint age–frailty composition of the cohort is projected as

(11) $$\overset{˜}{n}\left(t+1\right)=\overset{˜}{U}\overset{˜}{n}\left(t\right)$$

where the projection matrix is

(12) $$\overset{˜}{U}=DK^{T}UK\text{,}$$

with $K=K_{g,\omega }$ the vec-permutation matrix (Henderson and Searle, 1981, Hunter and Caswell, 2005, Caswell, 2012), which rearranges the population vector to permit multiplication by the appropriate block diagonal matrices. Because, in this special case, frailty is fixed, D is an identity matrix of dimension ωg×ωg and the formula reduces to

(13) $$\overset{˜}{U}=K^{T}UK\text{.}$$

### 3.2 Longevity: means, variances, and variance decomposition

The matrix $\overset{˜}{U}$ is the transient matrix of an absorbing Markov chain, with death as an absorbing state (e.g.,  Caswell, 2001, Caswell, 2009, Caswell, 2014). The fundamental matrix of this chain is

(14) $$\overset{˜}{N}={\left(I_{\omega g}-\overset{˜}{U}\right)}^{-1}$$

with dimension ωg×ωg. The (i,j) entry of $\overset{˜}{N}$ is the expected number of visits to state j by an individual in state i, where states include all combinations of age and frailty.

The statistics of longevity are calculated from $\overset{˜}{N}$ (e.g.,  Caswell, 2009). The vectors of first and second moments of longevity, and of the variance in longevity, are given by

(15) $${\overset{˜}{\eta }}_{1}={\left(1_{\omega }^{T}\overset{˜}{N}\right)}^{T}\;g\omega \times 1$$

(16) $${\overset{˜}{\eta }}_{2}={\left[{\overset{˜}{\eta }}_{1}^{T}\left(2\overset{˜}{N}-I\right)\right]}^{T}\;g\omega \times 1$$

(17) $$V\left(\overset{˜}{\eta }\right)={\overset{˜}{\eta }}_{2}-{\overset{˜}{\eta }}_{1}\circ {\overset{˜}{\eta }}_{1}\;g\omega \times 1\text{.}$$

These vectors contain the moments of the longevity of all gω ​age–frailty combinations. We are interested in the longevity of age class 1, which is a mixture of individuals with a mixing distribution π defined by the parameter λ that defines the variance of the gamma distribution. The vectors of means and variances of longevity in the g frailty groups within age class 1 are extracted from the full vectors by

(18) $$E\left(\eta_{\mathrm{groups}}\right)=\left(e_{1}^{T}⊗I_{g}\right){\overset{˜}{\eta }}_{1}\;g\times 1$$

(19) $$V\left(\eta_{\mathrm{groups}}\right)=\left(e_{1}^{T}⊗I_{g}\right)V\left(\overset{˜}{\eta }\right)\;g\times 1$$

where $e_{1}$ is a vector of length ω with a 1 in the first entry and zeros elsewhere.

The variance in longevity of age class 1, treated as a mixture of all the frailty groups with mixing distribution π, can be decomposed into a within-group component due to individual stochasticity and a between-group component due to heterogeneity in frailty:

(20) $$V\left(\eta \right)=E_{\pi }\left[V\left(\eta_{\mathrm{groups}}\right)\right]+V_{\pi }\left[E\left(\eta_{\mathrm{groups}}\right)\right]\;1\times 1$$

(21) $$=V_{\mathrm{within}}+V_{\mathrm{between}}\text{.}$$

The within-group component is the weighted mean of the entries of the vector $V\left(\eta_{\mathrm{groups}}\right)$:

(22) $$V_{\mathrm{within}}=\pi^{T}V\left(\eta_{\mathrm{groups}}\right)$$

(23) $$=\left(e_{1}^{T}⊗\pi^{T}\right)V\left(\overset{˜}{\eta }\right)\;1\times 1\text{.}$$

The between-group component is the weighted variance of the entries of the vector E($\eta_{\mathrm{groups}}$):

(24) $$V_{\mathrm{between}}=\pi^{T}\left[E\left(\eta_{\mathrm{groups}}\right)\circ E\left(\eta_{\mathrm{groups}}\right)\right]-{\left[\pi^{T}E\left(\eta_{\mathrm{groups}}\right)\right]}^{2}$$

(25) $$=\pi^{T}\left[\left(e_{1}^{T}⊗I_{g}\right){\overset{˜}{\eta }}_{1}\circ \left(e_{1}^{T}⊗I_{g}\right){\overset{˜}{\eta }}_{1}\right]-{\left[\left(e_{1}^{T}⊗\pi^{T}\right){\overset{˜}{\eta }}_{1}\right]}^{2}\;1\times 1\text{.}$$

This variance decomposition is a well-known theorem in probability theory (Renyi, 1970, Chapter 5.6, Theorem 1), forms the basis of the analysis of variance in statistics (Kempthorne, 1957), is used in quantitative genetics to calculate heritability (Falconer, 1975), and is widely used in the analysis of mixture models (Frühwirth-Schnatter, 2006).

The variance in longevity due to heterogeneous frailty is the variance among the mean longevities in the frailty groups. The variance due to stochasticity is the mean of the variances due to stochasticity within each frailty group. In the hypothetical situation in which survivorship is perfectly rectangular within each frailty group, $V_{\mathrm{within}}=0$ and all the variance in longevity is due to differences among the means. In the equally hypothetical situation in which the variance in frailty approaches zero, there is no heterogeneity and $V_{\mathrm{between}}=0$.

## 4 Parameter estimation

The gamma-Gompertz–Makeham model is fitted to cohort mortality data for the three selected countries.

### 4.1 Data (countries, cohorts, periods)

Cohort mortality data were obtained from the Human Mortality Database (http://www.mortality.org) for Italy (1872–1899), France (1816–1903), and Sweden (1751–1899). These countries were selected because of the availability of long mortality data times series of comparatively high data quality. The cohort mortality data consist of death counts (D(x)) and number of exposures (E(x)) at each age x for each birth cohort and for males and females separately.

To assure that our results were not dependent on the use of cohort survival, we also analysed period mortality data for Italy (1872–2012), France (1816–2013), and Sweden (1751–2014). Period mortality rates are calculated from data on deaths at each age in a specified year. The resulting mortality schedule applies to a ‘synthetic cohort’, an imaginary group of people who experience the demographic conditions in that year throughout their lives (Wilmoth, 2005). The ΓGM parameters estimated from period data apply to this synthetic cohort. As such, they more clearly reflect the impact of short-term mortality events, such as disease epidemics and wars, that affect mortality only in that period. On the other hand, period data do not reflect the patterns of real cohorts. As some demographers prefer cohort data and others prefer period data, we analysed both types of data to ensure that the choice of data did not influence our overall results. Also, both types of data appear in ecological studies; cohort data typically originating from laboratory longevity experiments and period data from capture–mark–recapture studies. Results for the period mortality analyses are given in the Appendix A.

### 4.2 Estimation of the ΓGM parameters

For each birth cohort, the four ΓGM parameters (i.e. a, b, c and γ) were estimated by maximum likelihood. Death counts D(x) were assumed to be Poisson-distributed with a rate parameter E(x)μ(x) (Brillinger, 1986), where E(x) denotes exposure at age x and the marginal hazard rate μ(x) depends on a, b, c and γ as in Eq. (3). The log-likelihood is

(26) $$\ln L\left[a,b,c,\gamma |D\left(x\right),E\left(x\right)\right]=\underset{x}{\sum }\left\{D\left(x\right)\ln\mu \left(x\right)-E\left(x\right)\mu \left(x\right)\right\}\text{,}$$

and was maximized by differential evolution (Storn and Price, 1997) using the R-package ‘DEoptim’ (Mullen et al., 2011). Differential evolution is a robust fast-converging global optimization method for possibly non-linear and non-differentiable continuous-space functions.

The ΓGM model was fit for a range of different starting ages for each cohort. Since the ΓGM model is often not considered appropriate to describe human mortality below age 40 and because above age 70, the number of exposed and deceased decrease fast, which could lead to unreliable estimates, we used starting ages 40, 50, 60 and 70.

## 5 Implementation of the age×frailty model

For each birth cohort and starting age, the estimates of a, b and c were used to create a baseline age-specific mortality schedule as in Eq. (4). The estimate of γ determines the variance in the initial gamma distribution of frailty. Two hundred frailty classes were defined, with a mean of 1, logarithmically spaced between a minimum and a maximum frailty value based on the cdf of the gamma distribution, such that $\text{cdf}\left(z_{\min}\right)=10^{-5}$ and $\text{cdf}\left(z_{\max}\right)=0.9999$. If the estimated variance of the frailty distribution was less than $10^{-5}$, heterogeneity was assumed to be zero and all individuals were assigned the frailty z=1. Mortality schedules were created with 150 age classes.

## 6 Results

**Fig. 1:**
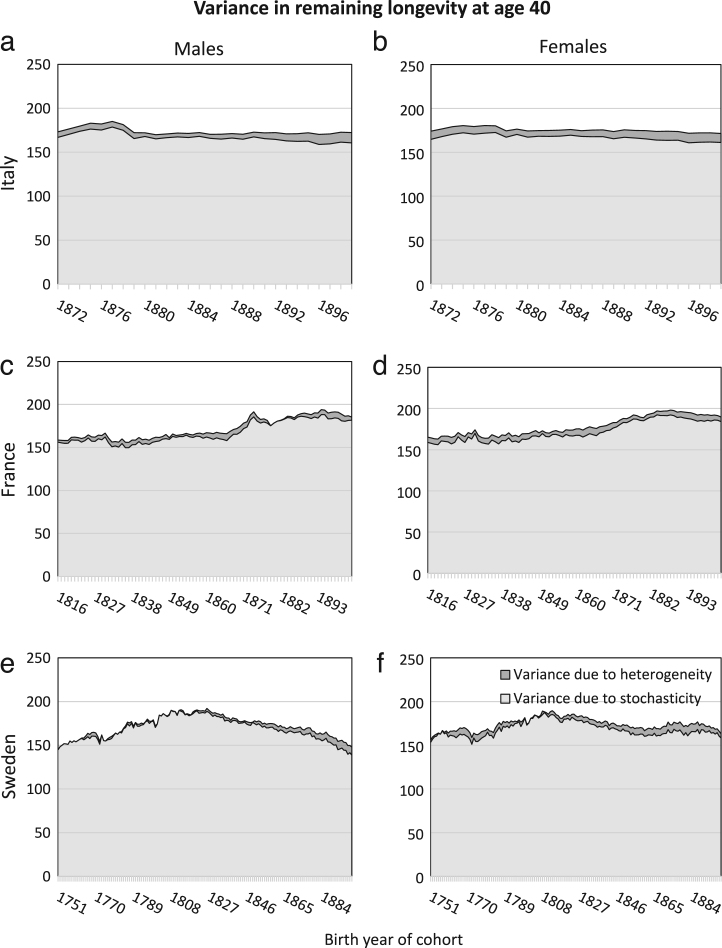
Variance in remaining longevity, conditional on survival to age 40, estimated from cohort mortality data. The variance is decomposed into variance resulting from stochasticity (light grey) and from heterogeneity (dark grey) and is plotted for all birth cohorts in the countries’ data set (1872–1899 for Italy, 1816–1903 for France and 1751–1899 for Sweden).

**Fig. 2:**
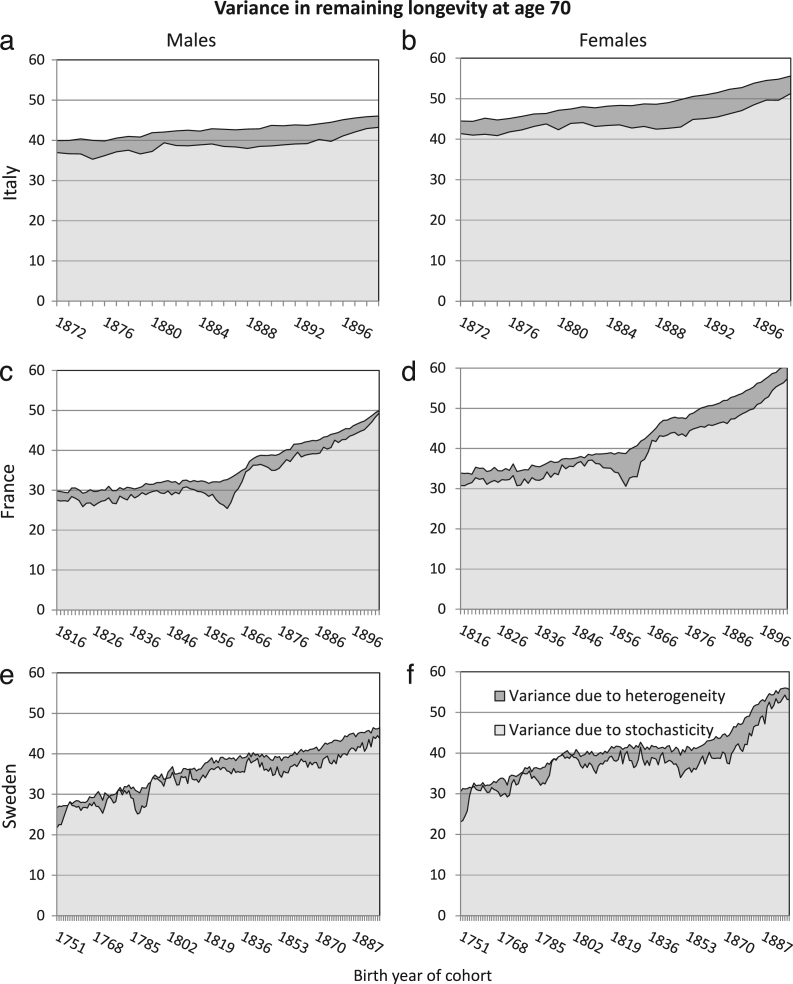
Variance in remaining longevity, conditional on survival to age 70, estimated from cohort mortality data. The variance is decomposed into variance resulting from stochasticity (light grey) and from heterogeneity (dark grey) and is plotted for all birth cohorts in the countries’ data set (1872–1899 for Italy, 1816–1903 for France and 1751–1899 for Sweden).

The patterns of variance in remaining longevity, conditional on survival to the starting age, are remarkably consistent across birth cohorts, sexes, countries, and starting ages (Figs. 1–2). As shown in Fig. 1, at starting age 40, the variance in longevity is $150\text{–}200\;a^{2}$, of which only 4%–5% is due to heterogeneous frailty. (The units of the variance are years squared.) At starting age 70, the variance in longevity is $30\text{–}60\;a^{2}$, of which 7%–10% is due to heterogeneous frailty (Fig. 2).

Averaging over cohorts within each of three historical periods (1751–1815, 1816–1871, and 1872–1899), we find that no more than 10% of the variance is due to heterogeneity, regardless of country, sex, or starting age. In each country and each historical period, the fraction of variance due to heterogeneity increases with starting age.

**Fig. 3:**
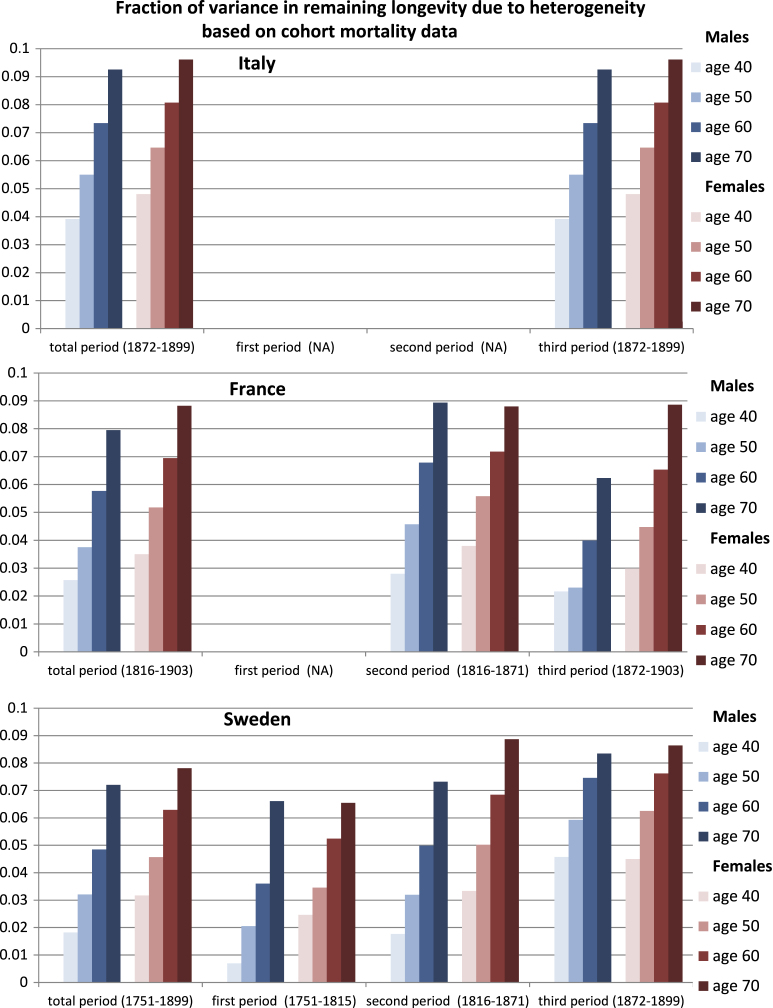
The mean fraction of the variance due to heterogeneity in remaining longevity for Italy, France and Sweden. Means are shown for the total period (1872–1899 for Italy, 1816–1903 for France, and 1751–1899 for Sweden) and also for three time periods: (1) 1751–1815, (2) 1816–1871, and (3) 1872–1899 (or 1872–1903, in the case of France).

The fraction of the variance that is attributable to heterogeneous frailty is shown in Fig. 3, in the form of a mean over birth cohorts, for starting ages of 40, 50, 60 and 70 years. Because cohort mortality data were available for different periods for each country, we show results for three time periods. During the first period (1751–1815), we have results only from Sweden; for the period 1816–1871, we have results for both Sweden and France; and for 1872–1899 (or 1872–1903, in the case of France), we have data for all three countries. In all cases, the fraction of variance due to heterogeneity increases with age. When considering the whole period, this fraction is below 0.10 for each country at age 70 and (much) lower for younger ages.

**Fig. A.1:**
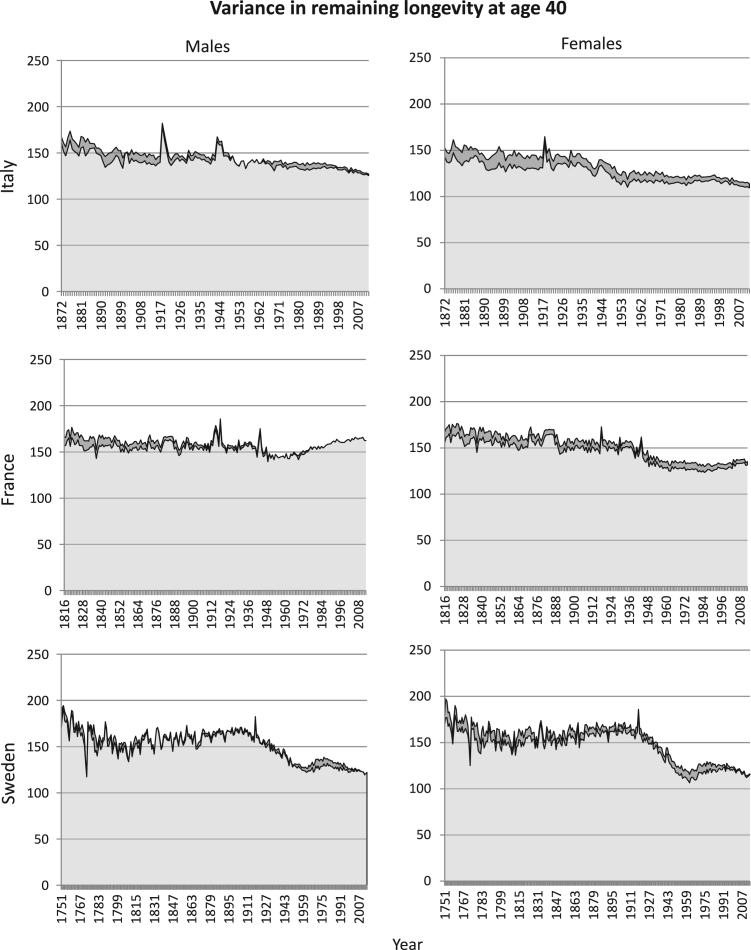
Estimated variance in remaining longevity, conditional on survival to age 40, based on the ΓGM model estimates obtained from period mortality data. The total variance is decomposed into variance resulting from stochasticity (light grey) and from heterogeneity (dark grey). The panels represent the results for males (left) and females (right) for Italy (1872–2012), France (1816–2013), and Sweden (1751–2014).

**Fig. A.2:**
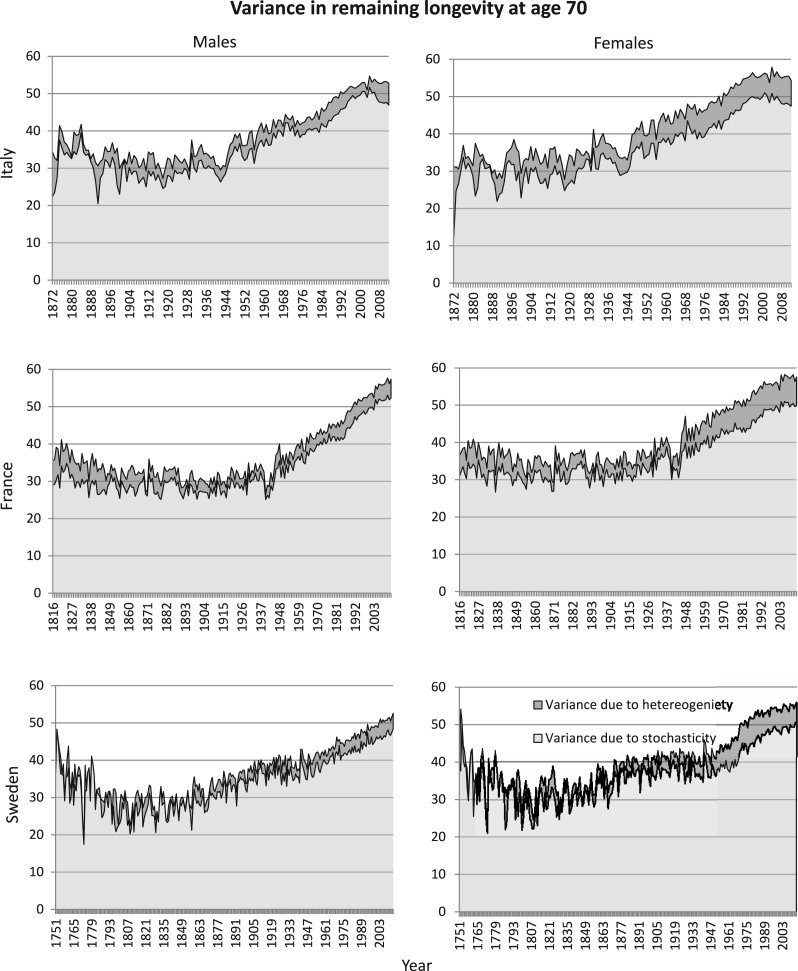
Estimated variance in remaining longevity, conditional on survival to age 70, based on the ΓGM model estimates obtained from period mortality data. The total variance is decomposed into variance resulting from stochasticity (light grey) and from heterogeneity (dark grey). The panels represent the results for males (left) and females (right) for Italy (1872–2012), France (1816–2013), and Sweden (1751–2014).

**Fig. A.3:**
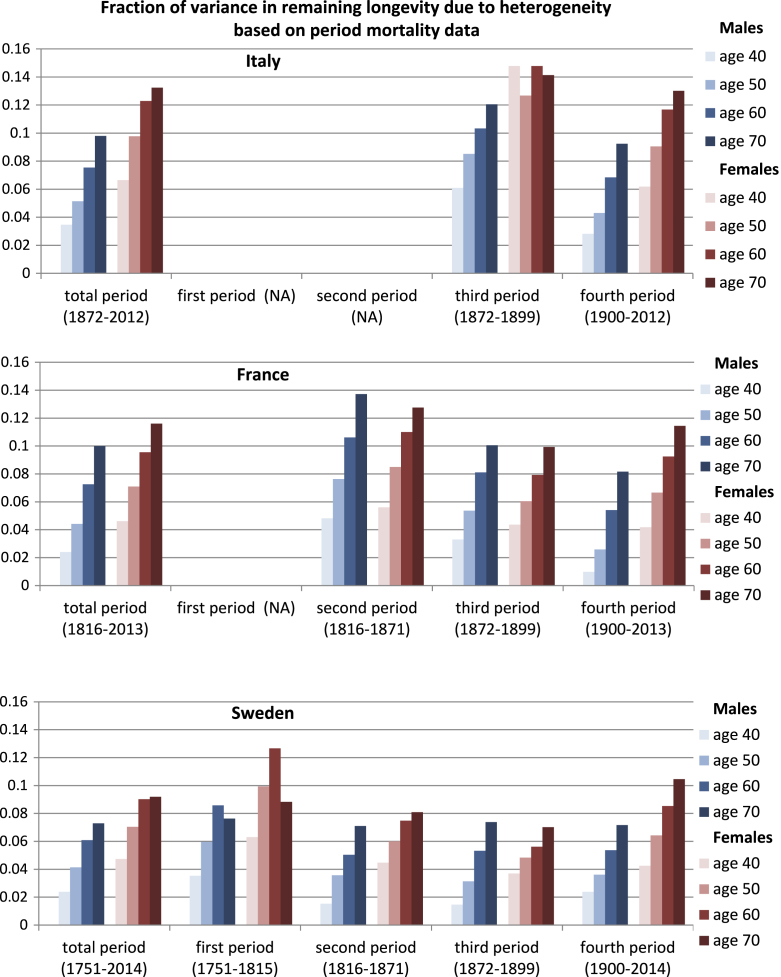
The mean fraction of variance, due to heterogeneity, in remaining longevity for Italy, France, and Sweden, based on ΓGM model estimates obtained from period mortality data. Means are shown for the total period (1872–2012 for Italy, 1816–2013 for France, and 1751–2014 for Sweden) and for the periods: (1) 1751–1815, (2) 1816–1871, (3) 1872–1899, and (4) 1900–2012 (or 2013 or 2014, for France and Sweden, respectively).

Analyses based on period mortality rather than cohort mortality permit us to examine more recent mortality patterns, although the analyses are of synthetic rather than real cohorts. The results (Fig. A.1, Fig. A.2, Fig. A.3) are similar to those for cohort data; the mean fraction of variance due to heterogeneity is never greater than 15%.

Although not central to our question, we note some interesting patterns in these data. First, the total variance in remaining longevity decreases with age; this is a well known property of human mortality schedules (e.g., Caswell, 2010). The variance in both cohort and period longevity at the oldest age examined here (70) increases in recent decades. The variance in period longevity at age 40 declines in recent decades, except for French males. These patterns are also well documented in other studies (e.g., Engelman et al., 2014).

## 7 Discussion

Individual stochasticity and heterogeneous frailty both contribute to the variance in longevity. Combining age (or stage) and frailty in a demographic model makes it possible to partition this variance into its components. Caswell (2014) presented a preliminary and somewhat ad hoc analysis of three published estimates of gamma-Gompertz and gamma-Siler models, and found that frailty contributed only 2%–7% of the variance. In this study, we have extended those results by analysing a large data set comprising cohort mortality data of three countries, using more rigorous estimation procedures. The long times series (28 years for Italy, 88 for France and 149 for Sweden for cohort data, and even longer series for period data) permit a more rigorous analysis of the relative contributions of stochasticity and heterogeneous frailty to the variance.

The results were consistent between countries and sexes: most of this variance in remaining longevity is due to stochasticity. Only a small fraction is attributable to heterogeneity. This fraction increases with starting age, because stochasticity-induced variance decreases faster with age than does heterogeneity-induced variance. However, even conditioning on survival to a starting age of 70 years, the average fraction due to heterogeneity is less than exceeded 0.10 (for cohort mortality) or 0.15 (for period mortality). Although data quality is, for obvious reasons, better for later cohorts and periods than for earlier ones, we found no clear temporal patterns in the fraction of variance due to heterogeneity. Only in the earliest period in Sweden, we see comparatively lower average values, which is due to the fact that the estimated heterogeneity in frailty was zero in some of the cohorts, probably due to the lower data quality in this period.

The ΓG and ΓGM models are widely applied to studies of human adult and late age mortality (Yashin et al., 2000, Horiuchi and Coale, 1990, Gage, 1989), but they make the strong assumption that heterogeneity is fixed and unchanging over the life of an individual. Dynamic heterogeneity occurs when individuals can change their state over time. Provided only that the dynamics are Markovian, dynamic heterogeneity can be incorporated into the matrix D in (12). The result is a multistate model incorporating age and, in this case, frailty (Caswell, 2014, Caswell, 2009, Caswell, 2012). Individual stochasticity, as in any demographic model, is then measured relative to the stages included in the model, and just as in fixed frailty models, can be decomposed into components within and between the heterogeneity classes. See Li and Anderson (2009) for a model based on a Wiener process for vitality, Caswell (2014) for a dynamic frailty model based on diffusion, and particularly Steinsaltz et al. (2012) for a valuable general discussion of the formulation and interpretation of mortality models in terms of Markov chains.

The estimates of the ΓGM parameters are affected by how well the model describes mortality, and how well the gamma distribution captures frailty (e.g.,  Heckman and Singer, 1982). The baseline Gompertz model assumes that mortality increases exponentially with age. In cohort data, the increase in age is confounded with the passage of time, and thus can be affected by short-term events that influence mortality. Such a mortality event may distort the estimation of the ΓGM parameters; high mortality rates at early ages may result in an overestimation of a, which may in turn affect the estimates of b, γ and c (Missov et al., 2015). For example, the small peak in total variance for French males of age 40 born in or around the year 1874 in Fig. 1(c) may be a result of this effect. For these men, the onset of the analysis coincides with the onset of World War I, which means that their cohort mortality data start with a few years of exceptionally high mortality rates. This effect may have created this peak visible for these cohorts, but it did not affect the order of magnitude of the variance nor the result that most of it is attributable to stochasticity.

Period mortality schedules are affected by short-term fluctuations, especially. There are, for example, small peaks in the total variance in longevity corresponding to the influenza pandemic of 1918, for both men and women, in Italy and France, at starting age 40 (Fig. A.1). No such peaks are apparent at starting age of 70 (Fig. A.2); the influenza pandemic particularly affected young adults. Our results from period mortality data are very similar to those from cohort mortality data. The estimated fraction of variance due to heterogeneous frailty is small for all countries and for all periods, for both men and women (Fig. A.3). Variance in longevity is mostly due to stochasticity rather than to heterogeneous frailty, independent of the type of mortality data used.

Note that we do not conclude that heterogeneity in frailty is generally unimportant, only that its contribution to the variance in remaining longevity is much less than that of individual stochasticity. Heterogeneous frailty has other effects not addressed here, such as the creation of mortality plateaus (Steinsaltz and Wachter, 2006, Missov and Vaupel, 2015).

Because models with unobserved heterogeneity are difficult to fit, and suitable data are not common, it is worth considering our results in an ecological perspective. Humans are long-lived, slowly developing, monovular large mammals. The data series we use represent a range of conditions sufficiently wide to change remaining life expectancy at age 40 by up to 50%, and at age 70 by up to 75%. Such effects in an animal population would be viewed as significant mortality changes. Across this range of conditions, and for both males and females, and for three populations, heterogeneity contributes only a small fraction of the variance in remaining longevity. Frailty (whatever may cause it) in human populations is, of course, expressed in the context of human social and cultural conditions. It will be interesting to compare these results with the components of variance in non-human species, in short-lived species, and over a range of field and laboratory conditions.

The vec-permutation matrix model for the joint age × frailty distribution is not limited to the Gompertz–Makeham mortality model or even to age-classified analyses (Caswell, 2014). It would apply equally well to models based on size, stage, or physiological state. Nor is it limited to the choice of gamma-distributed frailty, or to the case where the frailty is a fixed property of an individual. It applies equally well to other distributional choices, or to semiparametric finite mixture models of heterogeneity (Hartemink and Caswell, in preparation).

## Appendix A Period mortality data

See Fig. A.1, Fig. A.2, Fig. A.3.

## Appendix B Supplementary data

MMC S1 MATLAB scripts to calculate and decompose the variance in longevity for the matrix formulation of the gamma-Gompertz–Makeham model.
